# Long-term immunity after a single yellow fever vaccination in travelers vaccinated at 60 years or older: A 10-year follow-up study

**DOI:** 10.1093/jtm/taab126

**Published:** 2021-08-16

**Authors:** Mareen D Rosenstein, Adriëtte W de Visser, Leo G Visser, Anna H E Roukens

**Affiliations:** Department of Infectious Diseases, Leiden University Medical Center, Leiden, The Netherlands; Department of Infectious Diseases, Leiden University Medical Center, Leiden, The Netherlands; Department of Infectious Diseases, Leiden University Medical Center, Leiden, The Netherlands; Department of Infectious Diseases, Leiden University Medical Center, Leiden, The Netherlands

**Keywords:** Yellow fever vaccine, neutralizing antibodies, older travelers, waning immunity

## Abstract

**Background:**

In 2013, the World Health Organization (WHO) revised their position on yellow fever vaccination, in which revaccination every 10 years was no longer required, and that a single-dose provided life-long protection. However, research data on the immunogenicity of YF vaccine in people aged 60 years and over are scarce. Indeed, immunosenescence may result in lower virus neutralizing antibody titers after primary vaccination and a more rapid waning immunity. Therefore, we tested the hypothesis that older travelers, vaccinated at 60 years or older are more likely to become seronegative in comparison to young adults 10 years after primary YF vaccination.

**Methods:**

This is a 10-year follow-up study of an earlier prospective controlled cohort study. In the original trial, the neutralizing antibody response was measured in older travelers (aged 60–81 years, *N* = 28) and young adults (aged 18–28 years, *N* = 30) up to 28 days after a primary yellow fever vaccination. Ten years later, we collected serum samples of 22/28 (78%) elderly (71–85 years) and 14/30 (47%) controls (29–40 years), and determined their neutralizing antibody titers by plaque reduction neutralization test (PRNT_80_). Seropositivity was defined as plaque formation reduction of 80% at a serum dilution of 10 or more (PRNT_80_ ≥ 10).

**Results:**

All participants (36/36) were still seropositive 10 years after primary vaccination. The geometric mean concentrations were not statistically different between the older and younger participants (6.7 IU/mL vs. 8.6 IU/mL, *P* = 0.5).

**Conclusions:**

All older travelers were seropositive, 10 years after a primary YF vaccination at the age of ≥60 years. These data suggest that in older travelers a single vaccination is sufficient to convey long-lasting immunity for at least 10 years, and is in support the position of the WHO on a single-dose yellow fever vaccination.

## Introduction

Yellow fever is an acute hemorrhagic zoonotic disease caused by a flavivirus that is endemic in tropical regions of South America and sub-Saharan Africa. Transmission occurs through the bite of an infected mosquito, primarily by the species *Haemagogus* and *Aedes.*^1^ The course of the disease can be dramatic including severe liver damage, renal failure and hemorrhagic shock, with a case fatality rate of 20–60%.^1^

The live-attenuated 17D yellow fever vaccine has been in use for more than 70 years. It plays a crucial role in controlling the disease, especially since to date, no antiviral treatment is available.^1^ Vaccination has been proven safe and effective in evoking a robust and long-lasting immune response.^1^^,^^2^

Originally, a booster dose was recommended to all individuals every 10 years.^3^

In 2013, however, based on the recommendation of the Strategic Advisory Group of Experts (SAGE), the WHO revised its position on the use of yellow fever vaccination, and decided that a single dose confers sustained protection for the life of the person vaccinated, hence making revaccination no longer necessary.^4^ This change in policy was officially implemented in 2016,^5^ but has since caused scientific controversy as not all studies support the conclusion that a single vaccination provides lifelong protection,^6–9^ especially in certain groups such as young children, immunocompromised persons or persons of older age.

The demographic transition to an aging population is a global phenomenon. It is estimated that in 2050, nearly 17% of the world’s population will be aged 65 years and over.^10^ This also affects areas neighboring endemic countries without national routine immunization programs and at risk of yellow fever outbreaks, as well as the traveling population from non-endemic to endemic areas. These demographic changes further stress the necessity to gain more knowledge on the immunogenicity of a single-dose primary yellow fever vaccination at older age.

Persisting neutralizing antibodies have been found in older persons (≥60 years) more than 10 years after primary vaccination,^11–13^ however, these persons had received their first yellow fever vaccination at younger age (<60 years). More than 10 years ago, we investigated the immunogenicity of primary yellow fever vaccination at older age (60–74 years) and found a slower development of a neutralizing antibody response in comparison to younger persons (18–28 years).^14^ Similar results were found by a recent study by Casey et al., which showed that older persons (aged ≥50 years) had lower neutralizing antibody titers at 1 month after yellow fever vaccination compared to a group of younger persons (13–49 years).^15^ The results suggest different antibody kinetics in the two age strata, and as age is accompanied by immunosenescence, this can also impair the long-term effectiveness of vaccinations.^16^^,^^17^

In this study, we compared the seropositivity rates and geometric mean neutralizing antibody titers to yellow fever virus between older travelers (≥60 years) and younger controls (29–40) years, 10 years after primary vaccination.

## Methods

### Study design and participants

We performed a 10-year post vaccination follow-up study of an initial prospective controlled cohort study (2008–2009) (Dutch Trial Registry (DTR): NL1011). In the initial study (2008–2009), 28 older travelers, aged 60–81 years (median 66 years, IQR 65–69) and 30 younger controls, aged 18–28 years (median 21 years, IQR 20–23) were included. All study participants received a single dose of the 17D yellow fever vaccine from the same vaccine lot (Stamaril, Lot no B5355, Sanofi Pasteur, France). We found that elderly subjects had a delayed antibody response and higher viremia levels after primary yellow fever. After 1 month, all participants had attained a protective antibody response.^14^ All the participants from the previous study were eligible to participate, unless they had received an additional yellow fever vaccination between participation in 2008–2009 and the current study.

### Procedures

Blood specimen (8 mL) were collected during a short visit at the travel clinic of the LUMC or at the participant’s home. Samples were stored at −20°C.

In addition, information on the use of immunosuppressive drugs, on travel to flavivirus endemic countries within the last 10 years, and possible flavivirus-related disease were captured by questionnaire.

### Constant virus-varying serum dilution PRNT

The technique previously described by De Madrid and Porterfield^18^ was modified for the PRNT setup at Leiden University Medical Center (LUMC).^19^ Briefly, Vero cells were seeded in 6-well plates (Corning) and cultured until a monolayer was formed. Heat-inactivated sera were tested in serial 2-fold dilutions up to 1:512. Pooled seronegative sera were used as the negative control. One hundred plaque-forming units of 17D-YFV were added to each serum dilution. After an 1-hour incubation on ice, the mixtures of virus and serum were added to the Vero cell monolayers and incubated for 1 hour at 37°C; all were assayed in duplicate. An Avicel overlay was added. The overlay plates were incubated for 4 days at 37°C, followed by removal of the overlay and addition of formaldehyde (7%) for 60 minutes, which killed the virus and fixed the cell layer. After fixation, a 1-mL crystal violet solution was added for 10 minutes, staining only live cells. The plates were washed with distilled water and were dried for 1 day. The formed plaques were counted manually by two of the authors (AW and AR) who were blinded to the group allocation. Virus neutralization was calculated for each serum dilution as 100–100 × [(average number of plaques in the diluted post vaccination serum)/(average number of plaques in the negative controls)].

Seroprotection against yellow fever was defined as occurrence of 80% virus neutralization in a 1:10 or greater serum dilution.^20^ The serum end point titer was defined as the reciprocal of the serum dilution in which 80% virus neutralization occurred. End point titers were also reported in international units per milliliter according to the first International Reference Preparation for Anti-Yellow Fever Serum (National Institute for Biological Standards and Control).

### Primary endpoints

The primary endpoints were the proportion of seropositive individuals in each study group and the geometric mean concentration of yellow fever neutralizing antibodies in IU/mL.

### Statistical analysis

No formal sample size calculation was performed as the present study aimed at including as many former participants as possible. Categorical variables were described as counts and proportions, continuous variables as means along with the corresponding 95% confidence interval. Chi-square test was used to compare the proportion of seroprotection between the two groups. For the comparison of the geometric mean titer (GMT), an independent *t*-test was used. Statistical analysis was performed by SPSS, version 26.0 (IBM).

### Ethics

The study protocol was approved by the Ethics Committee Leiden The Hague Delft. The trial procedure was undertaken in accordance with Good Clinical Practice and complied with the Declaration of Helsinki. All participants provided written informed consent. The trial was registered under the Dutch Trial Registry number (NL8079).

## Results

**Figure 1:**
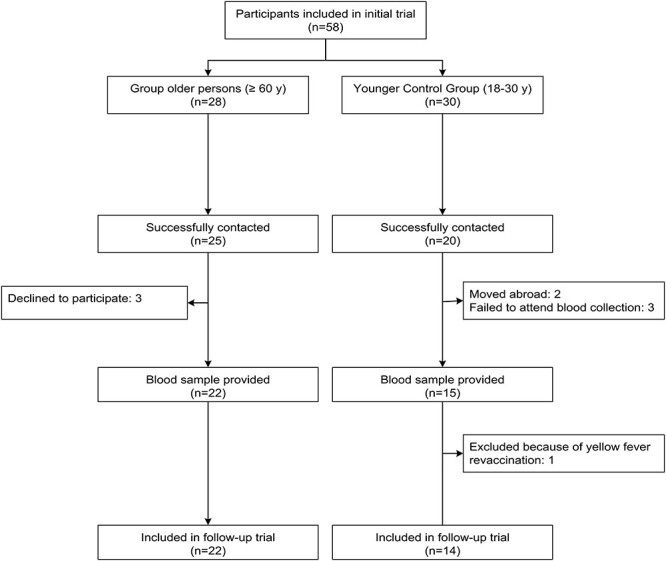
Flow chart of study participants *(no legend)*

Of the initial 58 participants, 45 were successfully contacted by the researchers, and of those, 36 volunteered to be enrolled in this follow-up extension study (Figure 1). One subject (younger control) had to be excluded because he had received a booster vaccination. However, none of the subjects had to be excluded because he or she received another flavivirus vaccination (i.e. Japanese encephalitis or tick-borne encephalitis).

The participants’ characteristics are given in Table 1. The median age was 77 years (IQR 73–79) and 33 years (IQR 32–34) in the older and younger study group, respectively. Of the whole study population, 75% were female. The mean time elapsed since the primary yellow fever vaccination for both groups was 10.9 years. In total, 4 subjects had travelled to a flavivirus endemic country within the last year but none had experienced any symptoms of a flavivirus infection.

**Table 1:** Characteristics of the participants

Characteristics	Group older persons (*n* = 22)	Group younger persons (*n* = 14)
Female, *n* (%)	17 (77)	10 (71)
Median age at primary vaccination (IQR), y	66 (65–69)	21 (20–23)
Reciprocal GMT at which 80% virus neutralization occurred 28 days post vaccination (95% CI)^a^	145 (96–219)	232 (127–425)
Median age at 10 years follow-up (IQR), y	77 (73–79)	33 (32–34)
Mean time since primary vaccination (range), y	10.8 (10–11)	11.0 (11)
Travel to a flavivirus endemic country in the last year^b^, *n* (%)	1 (4.5)	3 (21.4)
Travel to a yellow fever endemic country in the last ten years, *n* (%)	22 (100)	7 (50)
Flavivirus disease ^c^, *n*	0	0
Immunosuppressants^d^, *n*	0	0

^a^Determined in the initial trial

^b^Flavivirus endemic country as defined as endemic for Zika or yellow fever virus.

^c^Flavivirus disease as defined as yellow fever, Zika or Japanese encephalitis.

^d^Use of immunosuppressant drugs within the last 10 years.

GMT = geometric mean titer.

**Figure 2:**
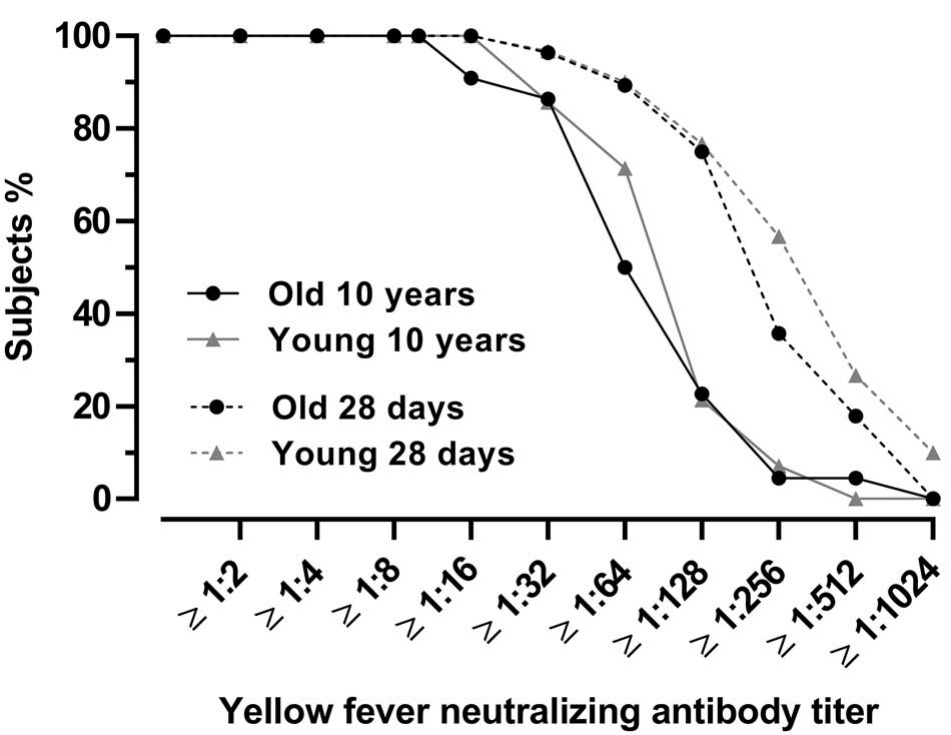
Yellow fever neutralizing antibody response in older participants compared to younger controls at 10 years and 28 days post-primary vaccination. Reverse cumulative distribution curves of yellow fever neutralizing antibody titers at 10 years after primary vaccination in 22 older and 14 young participants compared to 28 days post-primary vaccination of the former study (group older persons *n* = 28; younger group *n* = 30). Antibody titers represent the serum dilution at which 80% of virus was neutralized as assessed by PRNT_80_

All participants were seropositive, 10 years after primary vaccination (Table 2, Figure 2). The geometric mean concentration was slightly higher in the younger group as compared to the elderly group (+ 1.9 IU/mL, 95% CI, 0.4–1.5), but this difference was not statistically significant (*P* = 0.5). Mean reciprocal dilution at which 80% virus neutralization occurred, was 72 for the group of older subjects (95% CI, 47–108) and 85 for the younger subjects (95% CI 54–135) (Table 2). The reciprocal 80% neutralizing titers ranged from 12 to 751 (data not shown).

**Table 2:** Primary outcomes

	Group older persons (≥70 y) (*n* = 22)	Group younger controls (29–40 y) (*n* = 14)	*P*-value
Seropositivity^a^ (*n*, %)	22 (100)	14 (100)	-
Geometric mean concentration^b^ IU/mL (95% CI)	6.7 (4.4–10.2)	8.6 (5.3–14.0)	0.508
Reciprocal geometric mean serum dilution^c^ (95% CI)	85.1 (53.5–135.4)	71.6 (47.4–108.3)	0.511

^a^Defined as 80% virus neutralization (VN) in ≥1:10 dilution, measured by a PRNT_80_.

^b^Geometric mean concentration of yellow fever neutralizing antibody concentration in IU/mL.

^c^Geometric mean reciprocal serum dilution at which 80% of YF virus was neutralized.

Geometric mean yellow fever neutralizing antibody concentration and seropositivity rate among vaccinated elderly and younger controls assessed by PRNT_80_ at 10 years post-vaccination.

## Discussion

The most important finding of the present study was that all study participants, but especially the older participants (22/22) still had seroprotective antibody titers, 10 years after primary vaccination. The geometric mean titers were not significantly different between the group elderly and the younger subjects. This result rejects our hypothesis that older travelers vaccinated at 60 years or older might have a more rapid decay of yellow fever neutralizing antibodies.

In the present study, we observed in all older subjects long-lasting protective immune response after a single yellow fever vaccination, compared to the impaired immune response against other vaccines at older age. A possible explanation could be that the yellow fever vaccination is associated with a more pronounced systemic and longer lasting viral infection, which may drive the immunological response.^1^ Noteworthy, in the study of Casey RM et al., a fractional dose of the yellow fever vaccine (one fifth of the standard dose) evoked seroconversion in 98% (131/133) of all elderly participants (≥50 years) 1 year after primary vaccination.^15^

Another possible explanation is that this group of older travelers was a selection of persons of good mental health and physically active which may correlate with a younger biological age. They could only participate if they had an indication for yellow fever vaccination for travel purposes, implicating their good health.

A strength of the present study is the high follow-up rate in the group of older participants, as 22/28 (79%) of the initial study agreed to be included again in this follow-up research 10 years later.

Nevertheless, this study has a few limitations. First, only participants from the initial trial were eligible to participate in this follow-up study, which reduced the final sample size. A limitation of the small sample size is a reduced power, which impairs the ability to detect smaller differences between the two age groups that might really exist. Second, we were not able to include same numbers of volunteers in the two age-stratified groups as more younger participants had moved and we were not able to contact them, but the different sample sizes were accepted as this study focused on the outcomes of the older group. Third, of all participants, 33/36 (92%) subjects had travelled to a flavivirus endemic country within the last 10 years. We did not test for cross-reactive antibodies, and measured seropositivity due to the possibility of cross-reactivity cannot be completely excluded. Fourth, all elderly participants travelled to a yellow fever endemic country shortly after their primary yellow fever vaccination (2008–2009), versus only 7 (50%) of the younger participants in the last 10 years. An earlier boosting effect in the elderly travelers could therefore have impacted antibody decay rate. Finally, due to the limited sample size and the non-endemic location of the study, these results are based on laboratory findings and not on yellow fever infection rates.

In conclusion, this is the first study to be set up to evaluate the long-term immune response after a primary yellow fever vaccination in the vaccinated older traveler (≥60 years at vaccination). We found that all of the older subjects were seroprotected at 10 years post-vaccination. The results suggest that one single vaccination in the older traveler can establish long lasting immunity up to 10 years and is therefore in support of the decision of the WHO. However, larger studies, including studies in endemic areas are necessary to draw a generalized conclusion.

## Author contributions

M.R.: literature search, data collection, analysis and interpretation, writing of the manuscript.

A.V.: PRNT_80_, analysis of the data, revision of the manuscript.

L.V.: study design, literature search, interpretation of the data, revision of the manuscript.

A.R.: study design, data collection, literature search, data analysis and interpretation, revision of the manuscript.
